# What happens to your body during learning with computer-based environments? Exploring negative academic emotions using psychophysiological measurements

**DOI:** 10.1007/s40692-022-00228-w

**Published:** 2022-03-27

**Authors:** Kerstin Huber, Maria Bannert

**Affiliations:** School of Social Sciences and Technology, Department Educational Sciences, Chair for Teaching and Learning with Digital Media, Technical University of Munich, Arcisstr. 21, 80333 Munich, Germany

**Keywords:** Academic emotions, Electrodermal activity, Heart rate, Computer-based learning environments, Learning processes

## Abstract

This explorative study aims to examine if electrodermal activity (EDA) and heart rate (HR) are appropriate measures for identifying and monitoring academic emotions during learning in computer-based learning environments (CBLEs). Understanding learners' emotions while using CBLEs, allows improving the design of CBLEs. Therefore, we collected EDA, HR, and self-report data from 32 participants to measure academic emotions during learning with CBLEs in a laboratory setting. We induced negative academic emotions during learning using harmful connotated learning content about animal welfare. In a pre-post design, participants reported their emotional state before and after learning. We collated the self-reports with the EDA and HR curves to identify the emotional change in real-time. We prepared the data for repeated measurement analyses and group differences (high-, middle-, low learning performance; bored vs. not bored participants). Negative academic emotions were detected in increased EDA and HR. EDA turned out to be an indicator of learning performance. Boredom manifested in HR decrease. Findings show that EDA and HR are appropriate tools to measure academic emotions. We want to show the importance of real-time measures for learning and the efficiency of EDA and HR measures. It is worth considering EDA as a predictor for learning success and implementing EDA and HR measurements in CBLEs. However, more research is needed to clarify the role of HR in the context of learning performance.

**Supplementary Information:**

The online version contains supplementary material available at 10.1007/s40692-022-00228-w.

## Introduction

There is no doubt that emotions influence our learning behavior and outcome. When we are in a good mood, we learn more successfully (Arguel et al., 2017; Duffy et al., 2018; Loderer et al., 2020). This widely replicated insight shows that emotional states significantly impact learning performance (for a review, see Panadero, 2017; Loderer et al., 2020). Therefore, we considered it essential to explore emotions in the context of learning further.

Since we investigated computer-based learning environments (CBLEs), a specific set of emotions comes to the fore: emotions occurring in educational settings (e.g., studying at home, taking an exam, or being in class) are defined as *academic emotions* and are directly bound to learning and achievement. The most-reported academic emotions are anxiety, enjoyment, hope, pride, relief, anger, boredom, and shame (e.g., Duffy et al., 2018; Järvenoja et al., 2017; Loderer et al., 2020; Pekrun et al., 2002). Academic emotions are mainly evaluated post facto using self-report data (e.g., Boekaerts, 1999; Eteläpelto et al., 2018; Magno, 2011; Pekrun et al., 2011, 2017; Vermeer et al., 2000). However, a notable drawback of self-reports is that emotional states must be experienced consciously to report on them. Collecting post facto and self-report data reveals subjective responses about past events, which can cause measurement errors (e.g., Arguel et al., 2017; Laarni et al., 2015; Slater, 2002). Nevertheless, self-report data is a crucial and meaningful tool to gather subjective experiences, but it is limited according to an objective and implicit exploration of emotional processes during learning.

A promising approach to evaluate learning processes besides self-reports is “on-the-fly” measures stated by Winne and Perry (2000). Also, Järvelä and colleagues (2019) showed that analyzing real-time data is fruitful. They explored self-regulated learning by using qualitative content analyses, facial expressions, and psychophysiological measurements (i.e., electrodermal activity [EDA] and heart rate [HR]) in a collaborative learning setting. Confusion, for example, was detected based on a simultaneous increase in EDA, negative facial expressions, and a complimentary content analysis (Järvelä et al., 2019).

Our research goal is to provide deeper insights into learning (i.e., progression of the learning process besides self-reports, see section “Purpose of the study and research questions”) and explore the psychophysiological appearance of academic emotions. Based on the findings mentioned above and to balance the mentioned limitations of self-reports, the present study relied on psychophysiological measurements (i.e., EDA and HR) to examine academic emotions in CBLEs. Because changes in physiological behavior can have multiple reasons (see section “Psychophysiological measurements for academic emotions”), we eliminated as many confounding factors (e.g., the impact of social interactions in collaborative learning settings or movement artifacts) as possible by using a straightforward laboratory set-up. More precisely, we explored if specific physiological response patterns can be found, which indicate the current emotional state of the learner. Moreover, we seek to determine if physiological behavior can be a sufficient indicator for learning performance. Furthermore, we analyzed the change of academic emotions before and after stimulus presentation and whether this progress is evident in psychophysiological data.

## Theoretical framework

### The dual processing self-regulating model

The *Dual Processing Self-Regulating Model* from Boekaerts (2011) describes the essential role of emotions in learning. Boekaerts (2011) claimed that emotional states guide the learner's behavior onto one of two possible pathways. She proposed a *well-being* and a *growth pathway* as self-regulatory strategies, depending on how the task is assessed. Tasks that do not fit the current mental model trigger negative emotional states, which are detrimental for knowledge increase, leading the learner to take the well-being pathway. Tasks that correspond with the learner’s goals cause positive emotional states and thus open the growth pathway, resulting in knowledge increase. Measuring learner's emotional states can therefore propose a statement about learning success.

Furthermore, it is possible to switch from one pathway to the other. If learners are on the growth pathway and detect indicators for failing, they shift to the well-being pathway (Boekaerts, 2011). Determining this emotional shift in real-time enables immediate support and therefore guides the learner back on the growth pathway (see Arguel et al., 2017; D'Mello & Graesser, 2014). We want to find an appropriate “on-the-fly” measure that can identify negative emotional states during learning with CBLEs, as a step towards the primary goal of guiding and keeping the learner on the growth pathway.

### Academic emotions

Given that emotions are concomitants of learning, it is necessary to differentiate these academic emotions specifically (Pekrun & Stephens, 2012). Academic emotions, which can be seen in Table 1, are related to achievement, classroom settings, and learning. They are bound to success and failure, but also to the process of learning itself (Goetz & Hall, 2013; Pekrun et al., 2002, 2017). Multiple research approaches address academic emotions (e.g., confusion: D'Mello et al., 2014; boredom: Goetz & Hall, 2013; Pekrun, 2006; Pekrun et al., 2002). The underlying concept of this work is the *Three-Dimensional Taxonomy of Academic Achievement Emotions* from Pekrun (2006), which classifies academic emotions in three dimensions: their valence (positive or negative), activation (activating or deactivating), and object focus (activity or outcome; see Table 1). Enjoyment, for example, is, according to Pekrun (2006), a positive and activating academic emotion, during an activity (e.g., studying). In comparison, sadness is defined as negative and deactivating academic emotions triggered by pro- or retrospective failure (e.g., upcoming or past exams).

**Table 1:** A Three-dimensional taxonomy of academic achievement emotions

Object Focus	Positive^a^		Negative^b^	
	Activating	Deactivating	Activating	Deactivating
Activity	Enjoyment	Relaxation	Anger	Boredom
Outcome	Joy	Contentment	Anxiety	Sadness
	Hope	Relief	Shame	Hopelessness
	Pride		Anger	Disappointment
	Gratitude			

Academic Achievement Emotions categorized into three dimensions valence, activation, and object focus

^a^Positive = pleasant emotion

^b^Negative = unpleasant emotion (based on Pekrun & Stephens, 2012, p. 4)

In the psychophysiological literature, the term “arousal” is more common than “activation” (e.g., Berntson et al., 2017; Lang et al., 2009; Levenson et al., 2017; Potter & Bolls, 2012). To have consistent terminology in this article, we refer to the term “activation”.

Negative academic emotions usually trigger task-irrelevant thoughts and decrease the resources required for the task. Therefore, learning performance may decline if a learning goal seems unachievable due to prevalent negative academic emotions. However, negative activating academic emotions can also cause intense motivation to prevent failure, resulting in solving the task and increasing learning performance (Pekrun & Stephens, 2012). The shift from detrimental and conducive emotional states is also supported by Boekaerts’ *Dual Processing Self-Regulating Model* (2011; see chapter “Theoretical framework”), where learners switch from the well-being pathway to the growth pathway. Depending on the learner's assessment and the apparent solvability of a task, emotional states can change, and even knowledge can increase despite experiencing negative emotions during learning (Boekaerts, 2011).

Furthermore, task difficulty can affect academic emotions due to cognitive incongruity (Pekrun & Stephens, 2012). If the task seems too tricky or non-solvable, negative academic emotions are triggered, resulting in low learning performance (Baker et al., 2010; D'Mello & Graesser, 2014). Otherwise, positive academic emotions arise if a learning task can be solved, leading to high learning performance (Kang et al., 2008; Pekrun & Stephens, 2012).

In the present study, we decided to focus on negative activating academic emotions to reduce complexity. Besides, it is more valuable to properly understand the physiological appearance of negative academic emotions and cope with them to promote learning. We are interested in whether learners show an increase in knowledge despite the task causing negative academic emotions, or say it with Boekaerts’ approach if there is an increase in learning, a shift from the well-being to the growth pathway has happened.

### Psychophysiological measurements for academic emotions

Psychophysiological measures (e.g., EDA, electromyography, eye-tracking, or electrical activity of heart and brain) are well-elaborated to index cognitive tasks and emotional states (see Berntson et al., 2017; Dawson et al., 2017; Levenson et al., 2017). Psychophysiological measurements aim to conclude from physiological reactions to psychological processes (e.g., emotions or attention; Pinel & Pauli, 2012). Here, the essential statement is that physiological processes are intertwined with human behavior (Cacioppo et al., 2017). Based on psychophysiological data, conclusions concerning emotional processes can be drawn. Psychological conditions cannot be associated with a separate isolated physiological reaction. The complex reaction pattern must always be considered (Cacioppo & Tassinary, 1990). For example, an electrodermal reaction can indicate an arousing situation or a deep breath. Both situations show the same result—an increase in the electrodermal curve—but they are very different in their respective meaning. Therefore, there is no one-to-one relation between a single physiological response (e.g., an increase in EDA or HR deceleration) and a specific emotion (e.g., frustration). For example, an increase in EDA cannot identify frustration, and frustration does not express solely in changing EDA. Adding HR as a measure for valence can specify the increase in EDA since negative emotions express in HR decrease (see sections “Electrodermal activity” and “Heart rate”). Therefore, the psychophysiological pattern composed of EDA and HR curves must be considered to identify emotional states. The attribution from physiological response patterns to actual psychological meaning requires an accurate experimental design, appropriate data analyses, and interpretation (Cacioppo et al., 2017).

Since we see emotions as a two-dimensional model, both, valence and activation must be examined to capture emotions comprehensively. Then, merging EDA and HR data reveals a physiological pattern, which can identify emotional states (e.g., Barrett & Russell, 1999; Eteläpelto et al., 2018; Larsen & Diener, 1992; Levenson et al., 2017). Furthermore, only the valence can declare if the emotion is positive or negative, which is crucial for successful learning. We chose EDA and HR since these are easily measurable, non-invasive, sensitive to psychological states, and well-elaborated (see sections “Electrodermal activity” and “Heart rate”). Based on established research about psychophysiological measurements, we used EDA to capture the activation and HR to measure the valence of academic emotions. We do not further address the third dimension “object focus” because it refers to whether the emotional state is seen as activity or outcome (see Table 1), which is not relevant for our purpose.

#### Electrodermal activity

A standard psychophysiological measurement in many different research areas is EDA (e.g., attention, information processing, and emotion). Its popularity is the simple measurability and the sensitivity to many psychological states and processes (Dawson et al., 2017). EDA changes are associated with emotional activation, emotionally arousing thoughts or events, which induce an increase of electrical conductivity of the skin (Bradley, 2009). The EDA is solely controlled by the sympathetic nervous system (SNS) and, therefore, a direct reflection of activation (details see section “Heart rate”; Dawson et al., 2017; Lang et al., 2009). The interpretation of EDA changes depends on the stimulus material and the surroundings (Dawson et al., 2017). For example, an increase in EDA in an emotional surrounding can be interpreted as increased emotional activation. When somebody gets frightened, the increase in EDA can be traced back to the occurring attentional shift towards the unexpected stimulus (Bradley, 2009). Therefore, the more controlled a laboratory setting is, the more reliable is the interpretation of a change in EDA (Dawson et al., 2017). Moreover, having more than one measure (e.g., HR and self-reports) leads to a more accurate reconstruction of the learner's psychological state (Lang, 2014).

The most used method of recording EDA are skin conductance level and skin conductance response, both measured in microSiemens (μS). The tonic skin conductance level measures the conductivity of the skin in a particular situation and ranges from two to 20 μS. The phasic skin conductance response shows temporary fast changes in the conductivity of the skin caused by discrete events and ranges from one to five μS (Dawson et al., 2017).

#### Heart rate

Besides the primary function of pumping blood through the body, the heart also reveals information about emotion, attention, activation, and information processing (Berntson et al., 2017; Lang et al., 2009; Potter & Bolls, 2012). HR is, like EDA, easily measurable, non-invasive, and associated with many different psychological states. The HR shows the frequency of a cardiac cycle and is measured in beats per minute (bpm; Berntson et al., 2017). The most promising measurement is an inter-beat interval (IBI). Here, the time between two peaks of the cardiac cycle is tracked. The most prominent peak of the cardiac cycle is the R-spike. The time between two R-spikes is called RR-interval (Potter & Bolls, 2012).

Fluctuations in the HR can tell if a stimulus is pleasant or unpleasant, meaning HR is sensitive for measuring valence (Greenwald et al., 1989). Pictural stimuli (everyday objects or exciting scenes), which were assessed as pleasant (e.g., a beautiful landscape or erotic pictures), lead to HR acceleration, and pictural stimuli, assessed as unpleasant (e.g., dirty laundry or mutilated bodies), cause HR deceleration (Ijsselsteijn et al., 2000; Lang et al., 1993, 1997; Palomba et al., 1997). The valence of the pictural stimuli (pleasant or unpleasant) was evaluated and standardized by the International Affective Picture System, which can be used to explore emotion and attention (Lang et al., 1997).

Nevertheless, it is reasonable to assume that activating emotions lead to HR acceleration and deactivating emotions to HR deceleration. However, this relation does not necessarily persist based on the mechanics of the autonomic nervous system, which regulates HR and EDA. The link between activation and valence regarding the HR underlies the dual control of the heart. Its pace is regulated by both autonomic nervous branches, the parasympathetic nervous system (PNS), and the SNS (Berntson et al., 2017; Lang et al., 2009; Levenson et al., 2017). Both systems influence how fast the heart beats, depending on which system is activated. The activation of the PNS leads to HR deceleration, which is associated with attention and cognitive effort (Lang et al., 2009). The activation of the SNS results in HR acceleration, which is related to emotional activation (Lang, 1994). Therefore, HR can be a measure of valence but also activation. Nevertheless, since the PNS is faster and more dominant than the SNS, the activation of the SNS must be potent to overcome the parasympathetic activation (Shaffer & Ginsberg, 2017). A parameter to determine which system is activated is the heart rate variability (HRV), measured by spectral analyses (Berntson et al., 2017; Shaffer & Ginsberg, 2017).

### Purpose of the study and research questions

When we consciously experience emotions like love, happiness, anxiety, or distress, we feel our physiological reactions (e.g., faster heartbeat or sweaty hands). However, unconscious emotional states, especially in the context of learning, equally impact our physiological behavior and are thus detectable in psychophysiological curves. Furthermore, psychophysiology allows visualizing emotional processes in real-time (see section “Psychophysiological measurements for academic emotions”).

**Fig. 1:**
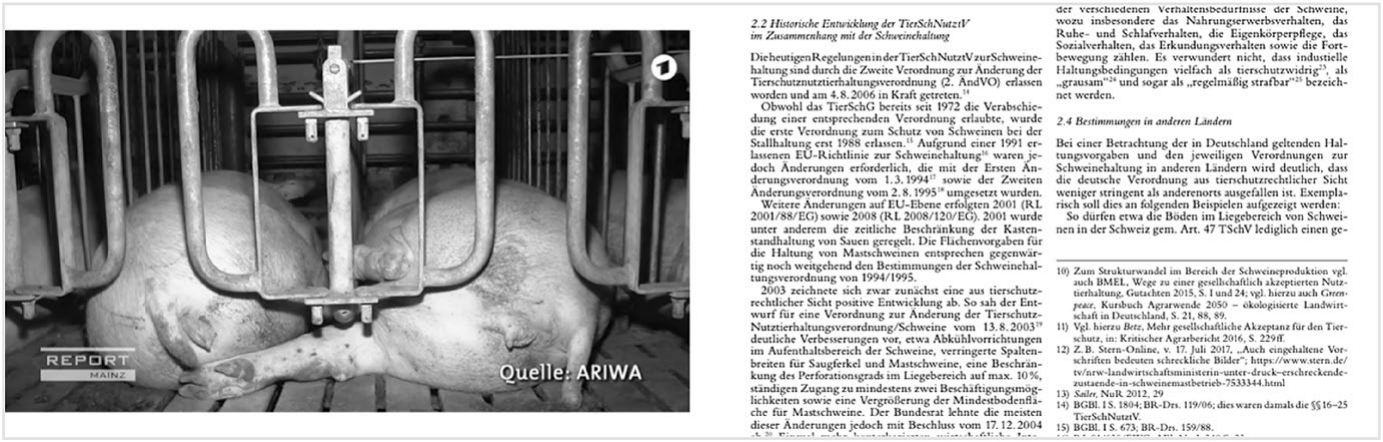
Screenshots of the Learning Material. Screenshot of the video on the left, an excerpt of the text on the right

Various studies have explored emotions in CBLEs and collaborative learning settings in a diverse manner (for a review, see Loderer et al., 2020). However, psychophysiological assessments of academic emotions in educational psychology are underutilized (Pekrun & Stephens, 2012). The present study wants to address this issue and get a unified and clear perspective on academic emotions, CBLEs, and self-reports. Moreover, we captured the valence and activation of academic emotions separately to give a detailed statement about the psychophysiological appearance of academic emotions. It was realized with a simple study design in a laboratory set-up (see Fig. 1) that eliminates potential external influencing factors (e.g., big-fish-little-pond effect; Preckel et al., 2008). The learning setting was designed to evoke negative emotions and guide the learner onto the well-being pathway. This process aims to be made physiologically detectable. Due to the lack of literature, the present work's research question and data analyses were primarily exploratory.

Since psychophysiological reactions unfold over time, they are an adequate measurement for academic emotions, which also occur over time. Self-reports give information about an emotional pre- and post-state of the learner – but they cannot provide details about the progression or reasons for the emergence of emotions. The exploratory research question (RQ) and hypotheses are structured top-down with the broad RQ at the top and the detailed hypotheses at the bottom. The derived RQ targets whether physiological behavior reveals more information about academic emotions and learning:

- Can psychophysiological measurements provide deeper insights into learning processes? The explorative character of the RQ allows space for different data analyses and approaches. The term “deeper insights” implies getting information about the ongoing learning process (psychophysiological data) rather than solely having information about the current state of knowledge (self-reports). Moreover, the cause, emergence, and physiological progression of academic emotions provide insights into learning behavior. We formulated detailed hypotheses to follow the top-down approach, referring to negative academic emotions and their physiological indicators. The hypotheses target specific data analyses to find distinct physiological patterns and thus indicators of academic emotions. We state that patterns in EDA and HR indicate negative academic emotions. To meet the requirements of the two-dimensional model of emotions, we formulate a particular hypothesis for each dimension. Valence is captured by HR, and EDA captures activation. Learning requires attention and information processing, which activates the PNS. In the psychophysiological context, this implies that the HR decreases. Moreover, the designed learning environment (see section “Learning environment”) included unpleasant stimuli, leading to HR decrease (see section “Heart rate”). Therefore, we state:
- Negative activating academic emotions cause HR deceleration over time (H1). Emotional activating situations cause an increase in EDA (see section “Electrodermal activity”). We want to show that this condition transfers to learning (i.e., academic emotions). The learning materials (see section “Learning environment”) induced negative activating academic emotions. Thus, we state:
- Negative activating academic emotions cause increasing EDA over time (H2). To associate learning, HR, and EDA, we formulated the third hypothesis. Task difficulty, analyzed using learning performance, has an impact on academic emotions (see section “Academic emotions”), which can be measured by changes in EDA and HR:
- Depending on the learning performance (high vs. low), overall HR and EDA differ (H3).

In conclusion, the *Dual-Processing Self-Regulating Model* (Boekaerts, 2011) shows that emotions have a crucial impact on learning (see chapter “Theoretical framework”). Since learners cannot always detect detrimental academic emotions, learning success can be affected negatively. We want to show an approach, which makes academic emotions measurable in real-time so that learners can be supported immediately. EDA and HR provide a fruitful measurement for emotions (see section “Psychophysiological measurements for academic emotions”). Based on the *Three-Dimensional Taxonomy of Academic Achievement Emotions*, we aim to measure both, valence and activation to distinguish between detrimental and beneficial academic emotions (the third dimension "object focus" has no further relevance for our approach). Anger and enjoyment, for example, are both activating but different in their valence. Only if both dimensions are measured, detrimental (e.g., anger) and beneficial (e.g., enjoyment) can be discriminated, and the learner can be supported accurately.

## Method

### Participants

Acquisition of participants was realized via a web-based online recruitment system *ORSEE* (Greiner, 2015). Participants were students and employees from the XXXX (*N* = 32; 21 females; *M*_*age*_ = 27.82, *SD* = 2.45). The inclusion criterion was being fluent in German to understand the stimulus material perfectly. We excluded one participant because of insufficient concentration and individual data channels with poor psychophysiological recordings. This results in different sample sizes for self-reports: *n* = 31 (20 females), HR: *n* = 28 (18 females), and EDA: *n* = 27 (16 females). Despite the small sample size, a sufficient test power (*β* = 0.80) according to an a-priori analysis (*α* = 0.05) can be achieved, which suggested 30 participants for mildly correlated repeated measures (*r* = 0.20) with a minimum of 16 number of measurements without baseline (Faul et al., 2009). Based on the mixed findings on whether emotions can be discriminated by indicating EDA and HR, we assume a medium effect size of *f* = 0.25 (Berntson et al., 2017; Boucsein, 2012; Levenson et al., 2017). Since we want to consider as much data as possible, we focused on the first 17 data points (incl. baseline), where all participants are included.

### Measures

#### Self-reports

We used the German versions of the Positive and Negative Affect Schedule (PANAS, Krohne et al., 1996; α ≥ 0.84; 5-point Likert-scale) and the seven-item short version of the Epistemically-Related Emotion Scale (EES-D, Pekrun et al., 2017; α ≥ 0.76; 5-point Likert-scale) in a pre-post design to measure the change of perceived emotional states after learning. We combined PANAS and EES-D because PANAS covers the overall emotional state (Krohne et al., 1996), and the EES-D refers to emotions accompanied by cognitive activities and knowledge generation (Pekrun & Stephens, 2012; Pekrun et al., 2017). Both questionnaires measure emotional activation and valence subjectively and are collated to EDA and HR as an objective measure for activation and emotional valence. The Academic Emotions Questionnaire (AEQ, Titz, 2001; α ≥ 0.84; 5-point Likert-scale) was only included in the posttest to retrieve information about the emotional experience of the previous learning situation. The AEQ consists of class-, learning-, and test-related emotion scales, which can be applied separately. Since we focus on the learning situation itself, we chose the learning-related emotion scale, which includes eight subscales (enjoyment, hope, pride, anger, anxiety, shame, hopelessness, boredom). Each item of the AEQ refers either to emotional experiences before, during, or after learning. To not overwhelm the participants, we used the 45 items of the AEQ, which gathered experiences during learning. The AEQ does not primarily refer to the valence or activation of emotions but mainly to the emotional evaluation of learning. Moreover, a short-form of a resilience scale (RS-13, Leppert et al., 2008; α = 0.69; 7-point Likert scale) was used before learning to determine possible correlations with emotional states and physiological behavior (prototypical items of the mentioned scales can be seen in Table S14 in the supplementary material). Learning performance was measured using a self-designed questionnaire with 10 multiple-choice items and one open question immediately before (prior knowledge) and after the learning session. (e.g., “*Conventional housing conditions for animals violate animal welfare laws. Why?*” or “*What is animal-turn?*” followed by four answer options). The score of the prior knowledge was subtracted from the score, which participants achieved after learning and is used to represent learning performance. To minimize guessing, participants always had the chance to mark “*I don't know*”. The open question queried a correct abbreviation for a technical term and was rated with one point for the correct spelling. Regarding the multiple-choice items, participants scored for marking the correct answer and not marking the incorrect answer with one point each, resulting in a maximum score of 33. All items refer to the content of the learning material, which measures knowledge increase after learning.

Consequently, the pretest contained PANAS and EES-D measuring the current emotional state, RS-13 gathering an unbiased value of resilience, and the content-related questionnaire testing prior knowledge. The posttest included PANAS and EES-D gaining the perceived change of emotional states, the content-related questionnaire measuring knowledge increase, and AEQ gathering the emotional experience of the previous learning situation. All scales and descriptive statistics for the present study can be seen in Table 2.

**Table 2:** Results for the self-report measures for negative emotions and scale-reliability

Measure		No. of items	Min	Max	*M*	*SD*	Cronbach’s α
PANAS^c^	Negative affect	10	1.07^a^	1.94^a^	1.30^a^	0.31^a^	0.747^a^
			1.39^b^	3.20^b^	2.36^b^	0.73^b^	0.877^b^
EES-D^c^	Confused, anxious, frustrated, bored	4	1.36^a^	1.52^a^	1.41^a^	0.49^a^	0.632^a^
			1.39^b^	2.36^b^	1.74^b^	0.59^b^	0.622^b^
AEQ^c^	Anger, anxiety, shame, hopelessness, boredom	32	1.74^b^	2.52^b^	2.12^b^	0.64^b^	0.868^b^
RS		13	4.68^a^	5.90^a^	5.23^a^	0.35^a^	0.687^a^
Learning performance^d^		11	6^a^	18^a^	11.7^a^	3.08^a^	–
			17^b^	26^b^	21.7^b^	2.48^b^	–

*N* = 31

^a^Pretest

^b^Posttest

^c^Itemized by valence

^d^Maximum score = 33

*PANAS* Positive And Negative Affect Schedule, *EES-D* Epistemically-Related Emotion Scale, *AEQ* Academic Emotions Questionnaire, *RS* Resilience Scale

#### Psychophysiological data

We used the BIOPAC MP36 system and the *Biopac Student Lab 4.1* software to record and process physiological data sampled with a 1 kHz rate. We sampled at a high rate to have valid data after smoothing and removing artifacts (see Boucsein et al., 2012). For proper measurements, we used the SS57L lead set and disposable snap Ag/AgCl pre-gelled electrodes for EDA and the fully shielded cable SS2LB with Ag/AgCl disposable snap pre-gelled electrodes EL501 for HR. From raw HR data, RR-intervals were derived in real-time for later analyses. Raw EDA data was treated with a 1 Hz FIR low-pass filter, and phasic data was derived from the tonic curve using a 0.05 Hz IIR high-pass filter. Artifacts were treated additionally with smoothing routines or interpolation methods. Furthermore, the baseline mean was subtracted from the curves to obtain standardized values and comparable data among all participants. The resulting channels with physiological data were resampled with 100 Hz and exported as text and excel files for further analyses.

The entire sequence of the study, that is, stimulus material, the participant's screen, and the recording of the participants—especially the placements of the electrodes, was recorded with *iMotions* version 8.1.

### Learning environment

We chose unpleasant stimuli as learning material to direct the participants on the well-being pathway and induce negative emotional states. The video is an actual report made by the public-sector broadcaster. The video consists of recordings made from animal welfarists in conventional pig farms and scenes of Germany’s political discussion about animal welfare. It starts with dramatic music and a voiceover, who reports about the illegally recorded scenes from pigsties, which were used to call attention to the mischief in conventional pig farming, triggering scare (see Fig. 1, on the left). Following scenes from a political event, the federal minister of Food and Agriculture (Germany) speaks about the danger that animal welfarists pose when recording illegally and that animals are protected by law. These scenes evoke an imbalance between reality and politics. Subsequently, the legal basis of conventional pig farming is presented. The conclusion is that many pig farms and the welfare of animals were not appropriately controlled, which activates anger. Then the illegally recorded scenes from pigsties continue, leading to sadness and distress. The voiceover continuously reports about the legal basis, the political discussion, and the animal protection act. The following scenes show how piglets were killed by an employee, which triggers distress and anger. The video concludes that the violation of the animal protection act is not punished sufficiently, resulting in frustration. Overall, the video induces severe negative emotions.

Afterward, the participants had to read a challenging scientific paper from Bruhn and Wollenteit (2018) about the detailed legal basis of the animal protection act and regulations. The text includes a lot of paragraphs and laws, which makes it difficult to read and understand (see Fig. 1, on the right). Because the participants were told to understand the content and recall as much information as possible, the task gets more difficult or even unsolvable, which should maintain the negative mood and lead to frustration and eventual boredom. The overall learning environment should affect the ongoing task appraisal in an emotionally negative manner, leading to perceived insolubility of the task. Therefore, a shift to the well-being pathway, indicated by changing psychophysiological behavior.

We pretested the learning material separately to ensure that it triggers negative emotions (*N* = 5). These pretests show that both stimuli evoke negative emotions (*p* < 0.05 for distressed, scared, hostile, upset, ashamed; detailed t-tests see Table S5 in the supplementary material).

### Procedure

Initially, we informed participants about the procedure of the study and psychophysiological data collection. We only shared the topic but no hypotheses or research interests. Then, participants had to sign a declaration of consent. Before the learning session started, participants answered questionnaires about resilience (RS-13), epistemic emotions (EES-D), current emotional states (PANAS), their political opinion about pig farming, eating habits, and prior knowledge about the topic to generate the learning performance score. During a rest period of five minutes, electrodes for the psychophysiological measurements were applied, which ensures an even hydration between the electrode, gel, and skin.

**Fig. 2:**
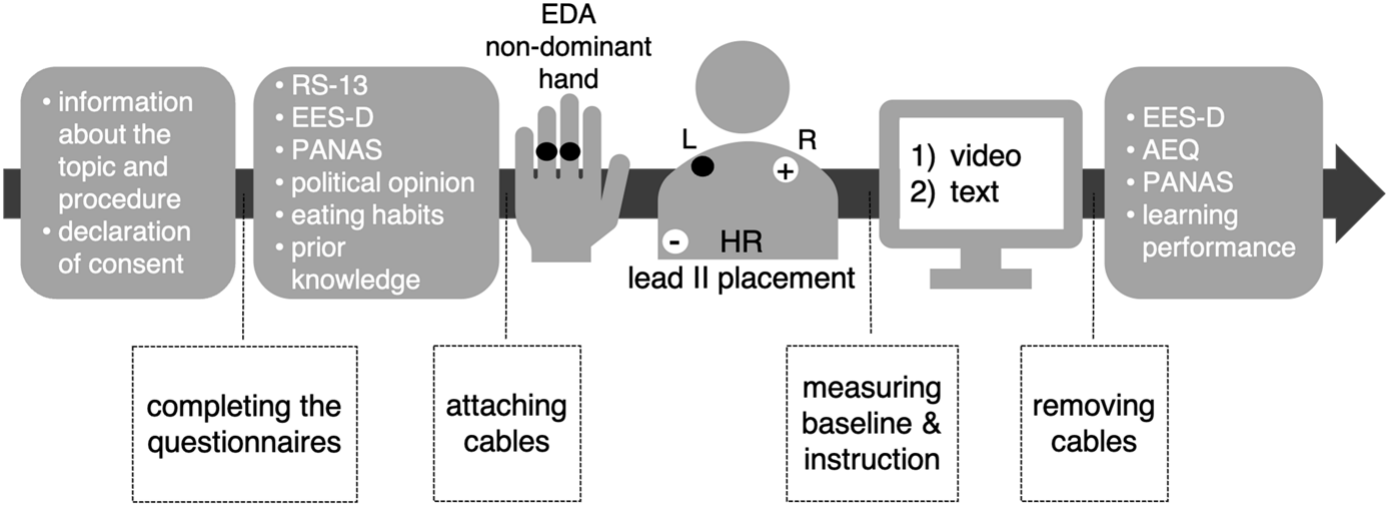
The Study Design, Including Every Step of the Procedure, all Instruments, and Placement of the Electrodes in Chronological Order From Left to Right

Moreover, the participants could get used to the laboratory set-up while a baseline was measured. Two electrodes were applied to the palmar proximal phalanges of the middle and ring finger of the non-dominant hand to record EDA. To collect HR data, we attached three electrodes according to the lead-II placement and the Einthoven Triangle to the upper body (two electrodes under the collarbone, one electrode on the left side of the ribcage, see Fig. 2). The learning session consisted of the six-minute video followed by the scientific text described above that started automatically after the baseline measurement using *iMotions* (version 8.1). We instructed the subjects to pay attention to the content and memorize as much information as possible immediately before the learning session. When the participants finished reading, cables and electrodes were removed. Afterward, information about the level of knowledge (learning performance), epistemic (EES-D), and academic (AEQ) emotions and current emotional state (PANAS) were gathered, and participants were informed about the research questions. The entire study lasted about one hour and took place in a laboratory of the XXXX.

### Data processing

All self-reports were collected using the online survey tool *SoSci Survey* and analyzed using *SPSS Statistics 26* (IBM Corp., 2020) and *JASP* (JASP Team, 2020).

The following treatments were recommended by the software creators (Sjak-Shie, 2019) and carried out in scientifically replicated standard procedures (see Boucsein et al., 2012; Cacioppo et al., 2017; Potter & Bolls, 2012).

**Fig. 3:**
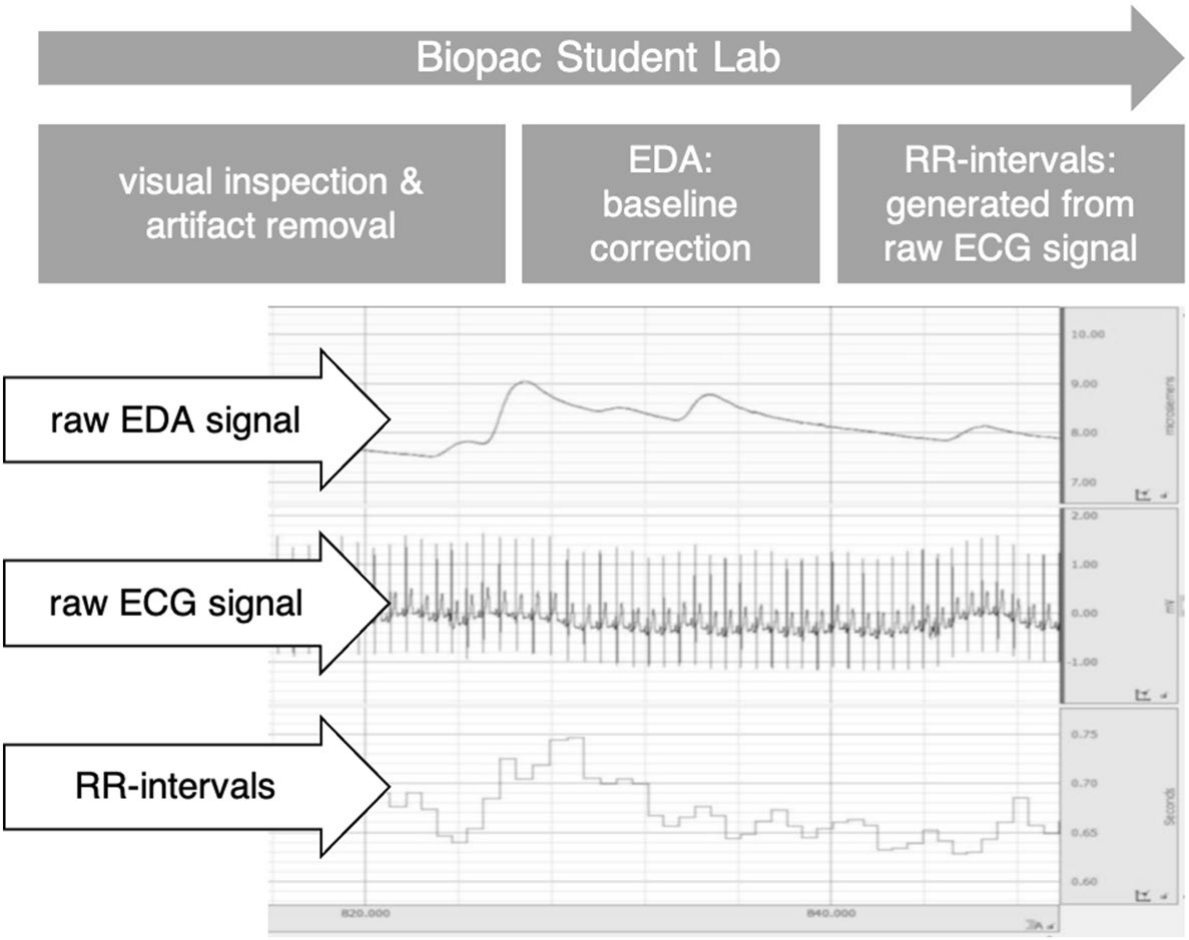
Chronological steps of data processing in the biopac student lab including a screenshot of data recordings. The displayed data stems from one of our participants

First, each data channel was checked visually for measurement errors or artifacts, and if necessary, smoothing or artifact removal procedures were used. The EDA signal was baseline corrected. The baseline correction is necessary because EDA can vary widely between and within participants (2 – 20 μS; see section “Electrodermal activity”) We subtracted the baseline, which was measured before the learning session (see Fig. 2) for each participant individually to generate comparable curves. The RR-intervals were generated in real-time from the raw electrocardiogram (ECG) using a standard procedure provided by *Biopac Student Lab*. Each step of data processing in the *Biopac Student Lab* and a screenshot of data recordings can be seen in Fig. 3.

**Fig. 4:**
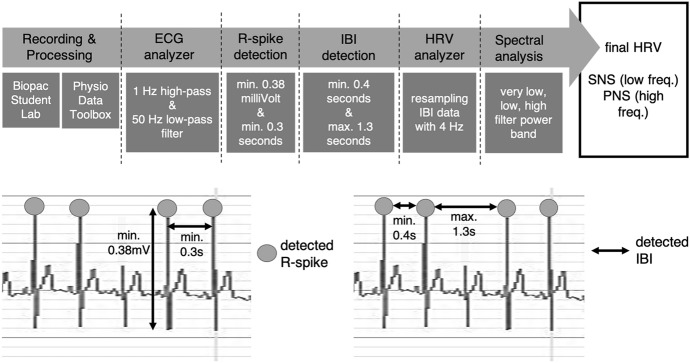
Illustration of Generating the Heart Rate Variability in the PhysioData Toolbox. Illustration of how R-spikes (on the Left) and Inter-Beat-Intervals (on the Right) Were Detected. The displayed data stems from one of our participants

To analyze HRV, we used the MATLAB-based application *PhysioDataToolbox* version 0.5 (Sjak-Shie, 2019). Therefore, the raw ECG signal was extracted from the *Biopac Student Lab*. The ECG signal analyzer treated the raw ECG data with a 1 Hz high-pass filter and a 50 Hz low-pass filter. To detect and count R-spikes, the minimum value of 0.38 millivolt and the minimum distance of 0.3 s between R-spikes must be fulfilled. Peaks below or above these values were not classified as R-spikes (see Fig. 4a on the left). Then, IBIs were derived from the detected R-spikes. A minimum value of 0.4 s and a maximum value of 1.3 s between the R-spikes must be fulfilled to be classified as IBI. IBIs with lower or higher values than these parameters were automatically rejected (see Fig. 4a on the right). The HRV analyzer used these generated IBIs and resampled them with a 4 Hz frequency. A spectral analysis was carried out to get information about which frequency components account for the variability of the heartbeat. Therefore, a very low (0.0033 Hz & 0.04 Hz), low (0.04 Hz & 0.15 Hz) and high (0.15 Hz & 0.4 Hz) filter power band were calculated. The resulting curves reveal whether the PNS (high-frequency) or the SNS (low-frequency) controls the heartbeat (see Fig. 4), which allows a proper interpretation of the HR data and their psychological meaning. The most descriptive output was the percentage distribution of each filter power band, and thus, if PNS or SNS controls the HR. The very low filter power band stands for thermoregulation, which is not relevant in our case.

After processing participants separately, we integrated all data in one file and visually lapped every data channel to identify outliers or abnormal curves between participants.

Finally, we exported all psychophysiological data in one excel-file for statistical analyses. We used the generated HRV data from *PhysioDataToolbox* and the data processed in *Biopac Student Lab* to analyze EDA, HR, and HRV data statistically.

We used two different methodical approaches. First, we prepared the data for repeated measurement analyses and group differences. Since we do not have specific areas or a stimulus onset but are interested in the progression of the curves over time, we averaged each data channel per minute, resulting in at least 17 (incl. baseline) values per participant (Min = 17, Max = 39; for HR: *M* = 27.0, *SD* = 5.54; for EDA: *M* = 27.1, *SD* = 5.64). These data segments were recommended by the software creators (Sjak-Shie, 2019). To avoid confusion: increasing HR represents decreasing RR-intervals.

To test H1 and H2, we conducted an ANOVA with repeated measurements to analyze if, when, or where psychophysiological curves differ. Therefore, we can explore how the curves progress over time. Most important when analyzing psychophysiological data is the visual inspection. Thereby, artifacts can be detected and removed easily. Afterward, an ANOVA with repeated measurements can be used as trend analysis. Here, the shape of the curves can be described. If a linear trend can be shown, the curves follow a linear progression. If the curves would fluctuate intensively, quadratic or cubic curves could be found, which is not to be expected in our case. Moreover, we conducted HRV analyses to determine which nervous system (i.e., PNS or SNS) controls the HR (see section “Heart rate”).

To test H3, we performed simple linear regression analyses, with EDA or HR as the predictor and learning performance as the dependent variable. Additionally, we performed a One-Way ANOVA to look for group differences in learning performance (high vs. middle vs. low).

It was noticeable that some participants were less bored after the learning phase than before. Therefore, we compared the psychophysiological curves of these participants to find patterns.

## Findings

### Self-reports

The presented results regarding emotional states stem from the EES-D, PANAS, and AEQ questionnaires.

All participants reported a significant increase in negative emotional states after learning (e.g., frustrated, distressed, scared, upset), indicating a negative appraisal of the task. However not significant, an unexpected tendency to a decrease in self-reported boredom after learning can be shown (see Table 3), which is in line with the verbal feedback from the participants. They expressed interest in the topic and wanted to receive more information.

**Table 3:** Self-report values for negative academic emotions and learning performance

	*M*_*pre*_	*SD*_*pre*_	*M*_*post*_	*SD*_*post*_	*t*(30)	*p*	Cohen's *d*
Frustrated	1.42	0.67	2.35	1.14	4.21	< 0.001	0.76
Distressed	1.55	0.85	3.00	1.07	6.86	< 0.001	1.23
Scared	1.06	0.25	3.13	1.38	8.58	< 0.001	1.54
Upset	1.13	0.34	3.19	1.20	8.92	< 0.001	1.60
Bored	1.52	0.77	1.39	0.56	−0.94	0.35	−0.17
Learning performance	11.7	3.08	21.7	2.48	13.2	< 0.001	2.37

*N* = 31

The learning performance was significantly higher in the posttest (see Table 3). Learning performance scores were normally distributed (Shapiro–Wilk test *p* = 0.95). Due to technical problems, poor psychophysiological data, or artifacts, the sample size varied. Detailed descriptive information can be found in the supplementary material.

### Psychophysiological data and learning performance

In the following sections, additionally, to test the hypotheses, exploratory analyses were carried out.

Simple linear regression analyses were used to examine whether psychophysiological behavior can predict learning performance. EDA data (i.e., the average skin conductance level) was used as a predictor and learning performance (i.e., difference score) as a dependent variable. The model showed a R^2^ of 0.27 (adjusted R^2^ = 0.24, *F*(1, 26) = 9.62, *p* = 0.005, *β* =  − 0.52), which indicated, according to Cohen (1988) a high goodness-of-fit. EDA was therefore a significant predictor for learning performance, *t*(27) =  − 3.10, *p* = 0.005. Regression analyses for HR data (i.e., average HR in bpm) did not show a convenient fit (*F*(1, 29) = 0.38, *p* = 0.54).

As exploratory analyses an ANOVA with repeated measurements was conducted using 60-s-slices for EDA and HR (see chapter 2.5.). EDA and HR curves followed a significant linear trend. EDA (*F*(1, 26) = 10.4, *p* = 0.003, η^2^_p_ = 0.29) and HR increased (*F*(1, 27) = 12.9, *p* = 0.001, η^2^_p_ = 0.32) significantly over 17 consecutive measuring points (i.e., 16 min incl. baseline T0; see Figs. 2 and 3). A significant difference of EDA (*F*(2.33, 60.5) = 8.91, *p* = 0.0002, η^2^_p_ = 0.26) and HR (*F*(5.26, 142) = 4.67, *p* = 0.0004, η^2^_p_ = 0.15) can be indicated with the highest increase in EDA after seven minutes into the experimental task (from *M* = 0.44, *SD* = 1.94 to *M* = 1.53, *SD* = 2.16; *t*(26) =  − 4.08, *p* = 0.0004; see Fig. 2, black dots) and the highest acceleration of the HR after six minutes into the experimental task (from *M* =  − 0.001, *SD* = 0.04 to *M* =  − 0.016, *SD* = 0.036; *t*(27) = 2.71, *p* = 0.012; see Fig. 3, black triangles; HR acceleration means decreasing RR-intervals).

**Fig. 5:**
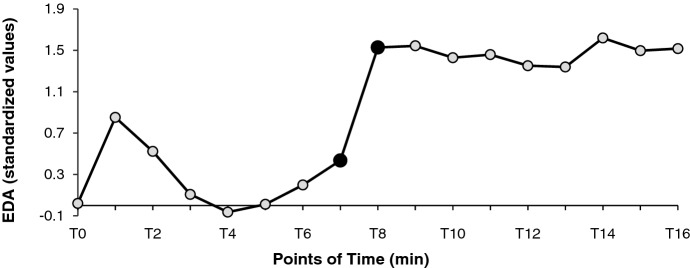
EDA Changes With the Most Significant Increase From T7 to T8 (Black Dots)

**Fig. 6:**
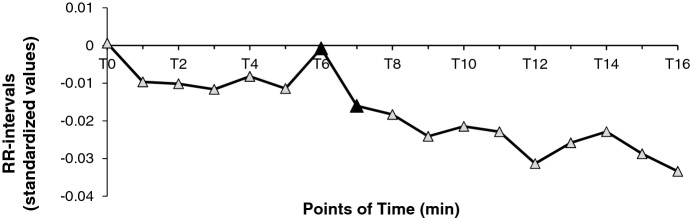
HR Changes, visualized in RR-Intervals, With the Highest Decrease from T6 to T7 (Black Triangles). Decreasing RR-Intervals Mean HR Acceleration

A distinctive feature can be observed after approximately six minutes (see Figs. 5 and 6): EDA and HR decline (i.e., RR curve rises) before increasing rapidly. At this time, the video ended and participants started reading the text, resulting in an attentional shift and a sudden increase in emotional activation (Lang, 2014). Afterward, the EDA and HR curves rose less sharply.

To examine whether the PNS or SNS controlled the HR, a spectral analysis for HRV was conducted using the *PhysioData Toolbox*. There, percentages were calculated to illustrate which nervous system was more active. The results showed that the low-frequency power (i.e., SNS) is 62.9 percent in charge of HR changes. At the same time, the high-frequency power (i.e., PNS) had only 29.8 percent control over HR. The remaining 7.23 percent corresponded to very low-frequency power and is associated with thermoregulation and, therefore, negligible.

**Fig. 7:**
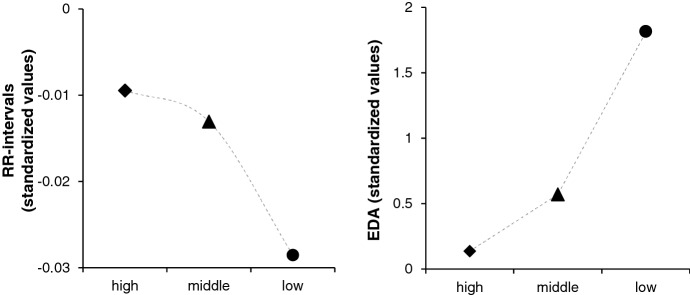
Groups of High, Middle, and Low Learning Performance to the Average of 17 Data Points of RR-Intervals and EDA. A Decreasing RR-Interval Curve Means HR Acceleration

Trend analyses showed a significant linear relation for EDA and learning performance (*F*(2, 24) = 4.10, *p* = 0.029, η^2^_p_ = 0.26) supported the finding that higher learning scores go along with low EDA (see Fig. 7, on the right). For HR data, no statistically significant relation to learning performance can be reported (*F*(2, 25) = 1.05, *p* = 0.37). However, a visual inspection showed a linear trend between decreasing HR and increasing learning performance (see Fig. 7, on the left). Therefore, we conducted a One-Way ANOVA, resulting in significant difference between the groups of high, middle, and low learning performance for HR (*F*(2, 25) = 52.6, *p* < 0.001, η^2^ = 0.81) and EDA (*F*(4.99, 59.9) = 2.30, *p* = 0.043, η^2^ = 0.161; descriptive information in Table 4).

**Table 4:** Groups of High, Middle, and Low Learning Performance for Electrodermal Activity (EDA) and Heart Rate (HR)

		*N*	*M*	*SD*
High	EDA	8	14.8	2.61
	HR	9	14.9	2.47
Middle	EDA	9	10.2	0.67
	HR	9	10.3	0.71
Low	EDA	10	5.60	2.22
	HR	10	5.60	2.22

**Fig. 8:**
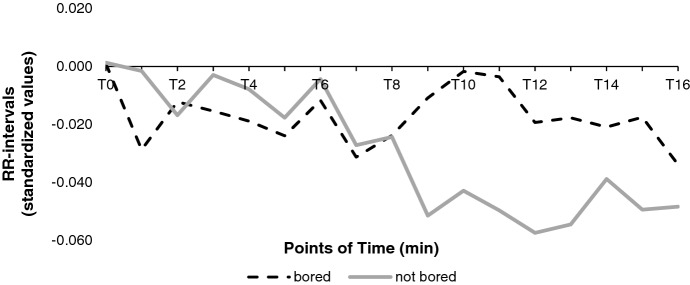
HR Comparison for Bored vs. not Bored Participants. From T8, the Curves Drift Apart. Not Bored Participants Show a Significantly Higher HR. Decreasing RR-Intervals Mean Increasing HR

As an explorative analysis, we compared participants which scored very high or very low on self-reported emotion questionnaires and analyzed whether specific emotions show a distinct psychophysiological pattern. Therefore, we aggregated the psychophysiological data of participants with differential values greater or less than zero (post–pre) individual items (sample size varies per item). Noticeable is the behavior in HR between bored (which scored one point higher in the posttest*; n* = 5) and not bored participants (which scored 2 points (*n* = 2) and one point (*n* = 5) lower in the posttest; see Fig. 8). Here, in four consecutive data points the HR was significantly higher for not bored (*n* = 7) than bored (*n* = 5) participants: after eight (*F*(1, 10) = 5.65, *p* = 0.039, η^2^ = 0.38), nine (*F*(1, 10) = 5.66, *p* = 0.039, η^2^ = 0.36), 10 (*F*(1, 10) = 6.06, *p* = 0.034, η^2^ = 0.38) and 12 (*F*(1, 10) = 5.16, *p* = 0.047, η^2^ = 0.33) minutes into the experimental task.

## Discussion and implications

In this work, we assessed if psychophysiological data can be used as an indicator for emotional states during learning with CBLEs and therefore predict learning performance (e.g., Pekrun & Stephens, 2012; Pekrun et al., 2011, 2017). Our exploratory research question targets the discourse of whether objective, real-time measures (i.e., psychophysiological data) reveal more information about the learning process than subjective post hoc self-reports. Compared to self-reports, which give the result of a learning session, psychophysiological data can measure what happens during the entire learning session and give real-time information about the learner's physiological behavior and emotional state. In our work, psychophysiological measurements were particularly fruitful given the progression of emotional states and task appraisal during learning and the shifting between the growth and well-being pathway. Different patterns were assessed by comparing groups that scored very high versus low on academic emotion scales. The characteristics, increasing EDA and HR, which interfere with learning, were detected. In addition, high EDA indicated low learning performance. Thus, psychophysiological measurements provide deeper insights into how and when academic emotions develop during learning than solely interpret self-reports.

Since our research question is relatively comprehensive, we defined precise hypotheses: Emotionally negative and activating learning material causes a decrease in HR and an increase in EDA, but differ depending on students’ learning performance (high, middle, low).

### Negative activating academic emotions cause HR deceleration over time (H1)

HR and negative activating emotions (frustration, distress, anxiety, and anger) increased after the learning phase, but this pattern is not aligned with our first hypothesis. However, our results indicate that the valence of deactivating academic emotions was expressed in HR because bored participants showed a lower HR (i.e., higher RR-intervals) than less bored participants (see Fig. 8). This leads to the assumption that HR can measure valence but not for highly activating emotions. Based on the research about to connection of HR and valence (see section “Heart rate”), HR can be a valid measure for valence. However, our learning environment's emotionally stimulating situation should be considered because the activation of the SNS could have superimposed the PNS and HR deceleration (Lang et al., 2009; see section “Heart rate”). This is in line with our finding that boredom expresses in decreasing HR (and increasing RR-intervals). Moreover, HRV analyses showed that the SNS is mainly in control over HR, concluding that the learning material was highly emotionally activating and therefore overcame the PNS (see section “Heart rate”). In summary, our first hypothesis cannot be supported, but the results indicate that changes in HR can reflect changing emotional states of learners.

A second possible explanation for the HR increase during the learning task besides high emotional activation of the learner can be the high cognitive load. Cranford and colleagues (2014) showed that tasks that cause a high cognitive load led to a higher increase in HR than tasks that elicit a small cognitive load. Also, Haapalainen and colleagues (2010) showed that ECG data was one of the most valuable indicators for cognitive load. Our results point in the same direction that HR displays rather cognitive load than the valence of academic emotions in a highly activating learning environment. Adding a control group with no emotionally activating stimuli would clarify this ambiguity. Moreover, qualitative data (open-ended questions or interviewing participants afterward) could provide a remedy.

### Negative activating academic emotions cause increasing EDA over time (H2)

EDA data followed a significant linear trend corresponding to HR data, which is, considering the self-report results, in line with our second hypothesis. Prior research (e.g., Eteläpelto et al., 2018; Kreibig, 2010) showed that high EDA values are indicators for emotionally high activation, which corresponds with our findings. Consequently, EDA can be used as a reliable measure for emotional activation during learning. However, EDA cannot determine the valence of academic emotions. Since activating and deactivating academic emotions can benefit learning, a measure for the valence is necessary. Herewith, the importance of measuring the valence of academic emotions becomes apparent. We showed that HR could not perform this task, at least in the context of learning. Therefore, more research is needed to identify a reliable indicator of valence for academic emotions.

### Depending on the learning performance, overall HR and EDA differ (H3)

Taking learning performance into account, a promising correlation can be found: With increasing EDA, the learning performance decreases. The activating learning material triggered negative academic emotions, which expressed in increasing EDA and led to poor knowledge increase. Though the posttest's learning score was significantly higher, the prior knowledge was relatively low due to the topic. So, it is not surprising that participants achieved a higher score in the posttest. Besides, the motivation of the learners could have been very high to prevent failure, resulting in high learning performance (see chapter 1.2.). This methodological issue should be considered for future research by choosing a more common topic. However, three significantly different groups for learning performance were identified. Therefore, EDA is a credible indicator of learning performance. For HR data, no clear statistical correlation was found. A trend can be detected when observing the results visually: with increasing HR, learning performance decreases. As a result, our third hypothesis can partly be supported.

Contrary to our expectations, the RR curve remains constantly below baseline level, triggered by activating, engrossing, and emotional learning material (Lang et al., 2009). This is in line with the findings that HR increases in highly emotional learning settings (Eteläpelto et al., 2018). Intense emotions like anxiety activate the SNS, resulting in faster HR and increasing EDA (Eteläpelto et al., 2018; Kreibig, 2010; Levenson et al., 2017). Based on these findings, the intensity of the experienced emotion could be the reason why we could not measure HR deceleration according to H3. We did not expect the overpowering emotional activation triggered by our learning environment. Our results point in the direction that in an emotionally high activating learning environment, HR is more sensitive for measuring cognitive load. Information input and attention usually go along with HR deceleration. When activating emotions, mental work, or concentration on inner thoughts are involved, the heart speeds up (Lang, 2014). This leads to the understanding that our setting provides an activating and emotional learning environment, which activates the SNS resulting in increasing EDA and HR. The activation of the SNS of our learning material overcomes the activation of the PNS, which slows the heart down (Lang et al., 2009).

An issue that remains to be discussed is the dramatic increase from T7 to T8 in EDA and from T6 to T7 in HR (see Fig. 5 and 6), which is a typical psychophysiological pattern for orienting responses. The reason behind an orienting response is the appearance of an unexpected stimulus (e.g., the sudden appearance of an error message on the screen or unexpected doorbell or call), which does not fit the current mental model (e.g., Bradley, 2009; Liebold et al., 2017; Potter & Bolls, 2012). This unexpected stimulus was the transition from the video to the text in our study. After the video stopped, the screen turned white before the text appeared. Moreover, the task shifted from watching the video passively to interacting with the input device (e.g., zooming the text in or out) and reading actively. Also, the participants' posture changed, from leaning back to sitting upright and closer to the screen. The EDA increased later than the HR because the electrodermal system is slower than the cardiovascular system (Berntson et al., 2017; Dawson et al., 2017). Since an orienting response refers to a short period and abates after a few seconds (Bradley, 2009), it has no further impact on our investigation.

Our overarching aim is to promote learning with CBLEs and find an implicit and real-time measurement for learning performance. Our research contributes to this issue by investigating how academic emotions manifest in psychophysiological data and validating physiological variables (e.g., EDA or HR) as a measurement of learning performance. The results are two options supporting the learner: as soon as a destructive academic emotion appears (e.g., frustration, boredom, anger (see Table 1), indicated by fluctuating and high EDA and HR), the learner receives support to solve the problem, prevent a switch to the well-being pathway (see chapter 1.1.), and lead the learner to learning success. The second assistance is identifying positive academic emotions (indicated by a steady EDA and HR), maintaining them, and keeping the learner on the growth pathway (see chapter “Theoretical framework”). Consequently, the learner’s individual needs can be considered without getting out of the flow (Arguel et al., 2017).

Our findings and prior research on the significance and performance of psychophysiological measures show that it is worth establishing these measurements in CBLEs. An early approach to assessing emotions via an input device was “The Emotion Mouse” (Ark et al., 1999), which has not gained further acceptance because of the intrusive hardware. Since the technical state of the art nowadays is more sophisticated (e.g., smartwatches, fitness, or activity trackers), it is simple and unobtrusive to include these devices in CBLEs.

## Limitations

Regarding our sample, gender differences can be noticed (see section “Participants”), which should be considered regarding the interpretation of the results of the self-reports. However, in a meta-analysis on emotions in technology-based learning environments, Loderer and colleagues (2020) could only find a weak relation between gender and academic emotions. Moreover, Frenzel et al. (2007) showed that gender had no direct effect on academic emotions. Consequently, despite the gender differences, our sample can be considered reliable. Due to drop-outs, no noteworthy gender differences resulted in physiological data.

Although the task triggered negative and learning-inhibiting academic emotions, learners showed a significant knowledge increase. However, attention should be paid to the low prior knowledge, ensuring a higher posttest score. In future studies, the content of the learning material should be considered to clarify further connections of EDA and HR with learning success. Identifying relevant areas turned out to be difficult as we cannot be sure that all learners read the same text passage simultaneously. Previously defined areas or controlled reading speed could counteract this issue. The resulting comparable sections are more manageable in data processing and interpretation than looking at the learning session overall.

More research is needed to determine psychophysiological patterns for successful learning processes besides emotional and activating learning environments. Furthermore, the technical implementation of psychophysiological measurements and processing in digital environments is uncertain.

## Conclusions

CBLEs gained importance, especially during the COVID-19 pandemic. However, learners' emotional states can hardly be identified by teachers in CBLEs. By making academic emotions measurable, learning progress can be better understood. The added value of this work is to comprehend the physiological appearance and impact of academic emotions on learning behavior and ultimately derive design approaches for CBLEs. In addition, this work aims further to validate psychophysiological measurements in the context of CBLEs, as this is relatively unattended (see Loderer et al., 2020).

Our findings show that psychophysiological measurements represent changes in academic emotions. Especially the distinction between the physiological behavior of bored and not bored participants can show shifting from Boekaerts’ (2011) growth to well-being pathway. Bored participants chose the emotionally deactivating well-being pathway, especially with the increasing duration indicated by lower HR.

In conclusion, we found the physiological pattern of increasing HR and EDA, which indicates negative activating emotional states of learners in academic settings and EDA as sufficient indicator for learning performance. However, self-reports are essential at this stage of research to identify individual emotional states. Based on our research, it is possible to head in the direction of promoting learning using psychophysiological measurements.

More research is needed to combine knowledge about the physiological emergence of emotions, the connection to the physiological appearance of academic emotions, and learning processes. Currently, these are rather separate research areas but would enormously benefit from each other. Moreover, qualitative data (e.g., interviews, open-ended questionnaires, or think-aloud data) can be included to extend the findings and contribute to the multimodal data approach (see Järvelä et al., 2019).

## Supplementary Information

Below is the link to the electronic supplementary material.

Supplementary file1 (DOCX 41 kb)

## Data Availability

On request.

## References

[CR1] Arguel A, Lockyer L, Lipp OV, Lodge JM, Kennedy G (2017). Inside Out: Detecting Learners' Confusion to Improve Interactive Digital Learning Environments. Journal of Educational Computing Research.

[CR62] Ark, W. S., Dryer, D. C., & Lu, D. J. (1999). The Emotion Mouse. In H. J. Bullinger & J. Ziegler (Eds.), *Human-Computer Interaction: Ergonomics and User Interfaces* (pp. 818–823). Lawrence Erlbaum Associates, Inc.

[CR2] Baker RSJ, d., D’Mello, S. K., Rodrigo, Ma. M. T., & Graesser, A. C. (2010). Better to be frustrated than bored: The incidence, persistence, and impact of learners' cognitive-affective states during interactions with three different computer-based learning environments. International Journal of Human-Computer Studies.

[CR3] Barrett LF, Russell JA (1999). The Structure of Current Affect: Controversies and Emerging Consensus. Current Directions in Psychological Science.

[CR4] Berntson, G. G., Quigley, K. S., Norman, G. J., & Lozano, D. L. (2017). Cardiovascular Psychophysiology. In J. T. Cacioppo, L. G. Tassinary, & G. G. Berntson (Eds.), *Handbook of Psychophysiology* (4th ed., pp. 183–216). Cambridge University Press. 10.1017/9781107415782.009

[CR5] Boekaerts M (1999). Motivated learning: Studying student* situation transactional units. European Journal of Psychology of Education.

[CR6] Boekaerts, M. (2011). Emotions, emotion regulation, and self-regulation of learning. In B. J. Zimmerman & D. H. Schunk (Eds.), *Handbook of Self-Regulation of Learning and Performance* (pp. 408–425). Routledge/Taylor & Francis Group.

[CR7] Boucsein W, Fowles DC, Grimnes S, Ben-Shakhar G, Roth WT, Dawson ME, Filion DL (2012). Publication recommendations for electrodermal measurements. Psychophysiology.

[CR8] Boucsein, W. (2012). *Electrodermal activity* (2nd ed). Springer.

[CR9] Bradley MM (2009). Natural selective attention: Orienting and emotion. Psychophysiology.

[CR10] Bruhn D, Wollenteit U (2018). Konventionelle Schweinehaltung und Tierschutzgesetz. Natur Und Recht.

[CR11] Cacioppo, J. T., Tassinary, L. G., & Berntson, G. G. (2017). Strong Inference in Psychophysiological Science. In J. T. Cacioppo, L. G. Tassinary, & G. G. Berntson (Eds.), *Handbook of Psychophysiology* (4th ed., pp. 3–15). Cambridge University Press. 10.1017/9781107415782.001

[CR12] Cacioppo JT, Tassinary LG (1990). Inferring psychological significance from physiological signals. American Psychologist.

[CR13] Cohen, J. (1988). *Statistical power analysis for the behavioral sciences* (2nd ed). Lawrence Erlbaum Associates, Inc.

[CR14] IBM Corp. (2020). *IBM SPSS Statistics for Windows* (26) [Computer Software].

[CR15] Cranford KN, Tiettmeyer JM, Chuprinko BC, Jordan S, Grove NP (2014). Measuring load on working memory: The use of heart rate as a means of measuring chemistry students' cognitive load. Journal of Chemical Education.

[CR16] D’Mello S, Lehman B, Pekrun R, Graesser A (2014). Confusion can be beneficial for learning. Learning and Instruction.

[CR17] Dawson, M. E., Schell, A. M., & Filion, D. L. (2017). The Electrodermal System. In J. T. Cacioppo, L. G. Tassinary, & G. G. Berntson (Eds.), *Handbook of Psychophysiology* (4th ed., pp. 217–243). Cambridge University Press. 10.1017/9781107415782.010

[CR18] D'Mello S, Graesser A (2014). Confusion and its dynamics during device comprehension with breakdown scenarios. Acta Psychologica.

[CR19] Duffy MC, Lajoie SP, Pekrun R, Lachapelle K (2018). Emotions in medical education: Examining the validity of the Medical Emotion Scale (MES) across authentic medical learning environments. Learning and Instruction.

[CR20] Eteläpelto A, Kykyri V-L, Penttonen M, Hökkä P, Paloniemi S, Vähäsantanen K, Eteläpelto T, Lappalainen V (2018). A multi-componential methodology for exploring emotions in learning. Frontline Learning Research.

[CR21] Faul F, Erdfelder E, Buchner A, Lang A-G (2009). Statistical power analyses using G*Power 31: Tests for correlation and regression analyses. Behavior Research Methods.

[CR22] Frenzel AC, Pekrun R, Goetz T (2007). Girls and mathematics —A "hopeless" issue? A control-value approach to gender differences in emotions towards mathematics. European Journal of Psychology of Education.

[CR23] Goetz, T., & Hall, N. C. (2013). Academic boredom. In R. Pekrun & L. Linnenbrink-Garcia (Eds.), *International Handbook of Emotions in Education* (pp. 311–330). Routledge/Taylor & Francis Group.

[CR24] Greenwald MK, Cook EW, Lang PJ (1989). Affective judgment and psychophysiological response: Dimensional covariation in the evaluation of pictorial stimuli. Journal of Psychophysiology.

[CR25] Greiner B (2015). Subject pool recruitment procedures: Organizing experiments with ORSEE. Journal of the Economic Science Association.

[CR26] Haapalainen, E., Kim, S., Forlizzi, J. F., & Dey, A. K. (2010) Psycho-physiological measures for assessing cognitive load. *Proceedings of the 12th ACM International Conference on Ubiquitous Computing* Doi: 10.1145/1864349.1864395

[CR27] Ijsselsteijn, de Ridder, H., Freeman, J., & Avons, S. E. (2000). Presence: Concept, determinants and measurement. Human Vision and Electronic Imaging.

[CR28] Järvelä S, Malmberg J, Haataja E, Sobocinski M, Kirschner PA (2019). What multimodal data can tell us about the students' regulation of their learning process?. Learning and Instruction.

[CR29] Järvenoja H, Järvelä S, Malmberg J (2017). Supporting groups' emotion and motivation regulation during collaborative learning. Learning and Instruction.

[CR30] JASP Team. (2020). *JASP* (0.12.2) [Computer software].

[CR31] Kang MJ, Hsu M, Krajbich IM, Loewenstein GF, McClure SM, Wang JT, Camerer CF (2008). The Wick in the Candle of Learning: Epistemic curiosity activates reward circuitry and enhances memory. SSRN Electronic Journal.

[CR32] Kreibig SD (2010). Autonomic nervous system activity in emotion: A review. Biological Psychology.

[CR33] Krohne HW, Egloff B, Kohlmann C-W, Tausch A (1996). Untersuchungen mit einer deutschen Version der „Positive and Negative Affect Schedule“ (PANAS). Diagnostica.

[CR34] Laarni, J., Ravaja, N., Saari, T., Böcking, S., Hartmann, T., & Schramm, H. (2015). Ways to measure spatial presence: Review and future directions. In Lombard M., Biocca F., Freeman J., IJsselsteijn W., Schaevitz R. (Eds.) *Immersed in Media*. Springer. 10.1007/978-3-319-10190-3_8

[CR35] Lang, A. (2014). *Measuring Psychological Responses To Media Messages*. Routledge.

[CR36] Lang PJ, Greenwald MK, Bradley MM, Hamm AO (1993). Looking at pictures: Affective, facial, visceral, and behavioral reactions. Psychophysiology.

[CR37] Lang PJ, Bradley MM, Cuthbert BN (1997). International affective picture system (IAPS): Technical manual and affective ratings. NIMH Center for the Study of Emotion and Attention.

[CR38] Lang, A., Potter, R., & Bolls, P. (2009). Where psychophysiology meets the media: Taking the effects out of mass media research. In J. Bryant & M. B. Oliver (Eds.), *Media Effects: Advances in Theory and Research* (3rd ed., pp. 185–206). Routledge.

[CR39] Lang, A. (1994). What can the heart tell us about thinking? In A. Lang (Ed.), *Measuring psychological responses to media messages* (pp. 111–124). Routledge/Taylor & Francis Group.

[CR40] Larsen, R. J., & Diener, E. (1992). Promises and problems with the circumplex model of emotion. In M. S. Clark (Ed.), *Emotion* (pp. 25–59). Sage Publications Inc.

[CR41] Leppert K, Koch B, Brähler E, Strauss B (2008). Die Resilienzskala (RS) – Überprüfung der Langfrom RS-25 und einer Kurzform RS-13. Klinische Diagnostik Und Evaluation.

[CR42] Levenson, R. W., Lwi, S. J., Brown, C. L., Ford, B. Q., Otero, M. C., & Verstaen, A. (2017). Emotion. In J. T. Cacioppo, L. G. Tassinary, & G. G. Berntson (Eds.), *Handbook of Psychophysiology* (4th ed., pp. 444–464). Cambridge University Press. 10.1017/9781107415782.020

[CR61] Liebold B, Brill M, Pietschmann D, Schwab F, Ohler P (2017). Continuous measurement of breaks in presence: Psychophysiology and orienting responses. Media Psychology.

[CR43] Loderer K, Pekrun R, Lester JC (2020). Beyond cold technology: A systematic review and meta-analysis on emotions in technology-based learning environments. Learning and Instruction.

[CR44] Magno, C. (2011). Validating the Academic Self-regulated Learning Scale with the Motivated Strategies for Learning Questionnaire (MSLQ) and Learning and Study Strategies Inventory (LASSI). *The International Journal of Educational and Psychological Assessment*, *7*.

[CR45] Palomba D, Angrilli A, Mini A (1997). Visual evoked potentials, heart rate responses and memory to emotional pictorial stimuli. International Journal of Psychophysiology.

[CR46] Panadero E (2017). A Review of Self-regulated Learning: Six Models and Four Directions for Research. Frontiers in Psychology.

[CR47] Pekrun R (2006). The Control-Value Theory of Achievement Emotions: Assumptions, Corollaries, and Implications for Educational Research and Practice. Educational Psychology Review.

[CR48] Pekrun R, Goetz T, Titz W, Perry RP (2002). Academic Emotions in Students' self-regulated learning and achievement: a program of qualitative and quantitative Research. Educational Psychologist.

[CR49] Pekrun R, Goetz T, Frenzel AC, Barchfeld P, Perry RP (2011). Measuring emotions in students' learning and performance: The Achievement Emotions Questionnaire (AEQ). Contemporary Educational Psychology.

[CR50] Pekrun R, Vogl E, Muis KR, Sinatra GM (2017). Measuring emotions during epistemic activities: The Epistemically-Related Emotion Scales. Cognition and Emotion.

[CR51] Pekrun, R., & Stephens, E. J. (2012). Academic emotions. In K. R. Harris, S. Graham, T. Urdan, S. Graham, J. M. Royer, & M. Zeidner (Eds.), *APA Educational Psychology Handbook, Vol 2: Individual Differences and Cultural and Contextual Factors* (pp. 3–31). American Psychological Association. 10.1037/13274-001

[CR52] Pinel, J. P. J., & Pauli, P. (2012). *Biopsychologie* (8th ed.). Pearson, Higher Education.

[CR53] Potter, R. F., & Bolls, P. (2012). *Psychophysiological Measurement and Meaning: Cognitive and Emotional Processing of Media*. Routledge/Taylor & Francis Group.

[CR54] Preckel F, Zeidner M, Goetz T, Schleyer E (2008). Female "big fish" swimming against the tide: The "big-fish-little-pond effect" and gender ratio in special gifted classes. Contemporary Educational Psychology.

[CR55] Shaffer F, Ginsberg JP (2017). An Overview of Heart Rate Variability Metrics and Norms. Frontiers in Public Health.

[CR56] Sjak-Shie, E. E. (2019). *PhysioData Toolbox* (0.5) [Computer software]. https://PhysioDataToolbox.leidenuniv.nl

[CR57] Slater M (2002). Presence and The Sixth Sense. Presence Teleoperators and Virtual Environments.

[CR58] Titz, W. (2001). *Emotionen von Studierenden in Lernsituationen: Explorative Analysen und Entwicklung von Selbstberichtskalen*. Waxmann.

[CR59] Vermeer HJ, Boekaerts M, Seegers G (2000). Motivational and gender differences: Sixth-grade students' mathematical problem-solving behavior. Journal of Educational Psychology.

[CR60] Winne, P. H., & Perry, N. E. (2000). Measuring self-regulated learning. In M. Boekaerts, P. R. Pintrich, & M. Zeidner (Eds.), *Handbook of Self-Regulation* (pp. 531–566) Academic Press. 10.1016/B978-012109890-2/50045-7

