# Supplementary material for: What happens to your body during learning with computer-based environments? Exploring negative academic emotions using psychophysiological measurements
Source: J Comput Educ. 2022 Mar 27;10(1):189–215. doi: 10.1007/s40692-022-00228-w (PMC8958339; doi:10.1007/s40692-022-00228-w)
Supplement: Supplementary file 1 — Supplementary file1 (DOCX 41 kb) [file 40692_2022_228_MOESM1_ESM.docx]

**Supplementary Material**

**Results for the pretest to ensure that the learning material triggers negative emotions.**

**Table 5**

*Results of the Pretest for the Learning Material using the PANAS Questionnaire*

| PANAS | *M* | | *SD* | | *t*(4) | | *p* | |
| --- | --- | --- | --- | --- | --- | --- | --- | --- |
|  | text | video | text | video | text | video | text | video |
| active (aktiv) | .000 | .000 | 1.225 | 1.581 | .000 | .000 | 1.000 | 1.000 |
| interested (interessiert) | - .400 | -.400 | 1.342 | .894 | -.667 | -1.000 | .541 | .025 |
| excited (freudig erregt) | -1.800 | -1.400 | 1.789 | 1.140 | -2.250 | -2.746 | .088 | .052 |
| strong (stark) | -1.400 | -.800 | 1.342 | 1.095 | -2.333 | -1.633 | .080 | .178 |
| inspired (angeregt) | .600 | -.800 | 2.074 | 1.304 | .647 | -1.372 | .553 | .242 |
| proud (stolz) | -1.800 | -.800 | 1.304 | .837 | -3.087 | -2.138 | .037 | .099 |
| enthusiastic (begeistert) | -1.800 | -1.200 | 1.643 | 1.304 | -2.449 | -2.058 | .070 | .109 |
| alert (wach) | .400 | -.400 | .548 | .548 | 1.633 | -1.633 | .178 | .178 |
| determined (entschlossen) | .000 | -.600 | 1.000 | 1.342 | .000 | -1.000 | 1.000 | .374 |
| attentive (aufmerksam) | .000 | .400 | 1.414 | .894 | .000 | 1.000 | 1.000 | .374 |
| distressed (bekümmert) | 1.800 | 1.400 | 1.095 | .894 | 3.674 | 3.500 | .021 | .025 |
| upset (verärgert) | 2.000 | 1.600 | 1.225 | 1.517 | 3.651 | 2.359 | .022 | .078 |
| guilty (schuldig) | 1.400 | 1.400 | 1.140 | 1.342 | 2.746 | 2.333 | .052 | .080 |
| scared (erschrocken) | 1.400 | 2.200 | 1.140 | 1.643 | 2.746 | 2.994 | .052 | .040 |
| hostile (feindselig) | .400 | 1.200 | 1.342 | .837 | .667 | 3.207 | .541 | .033 |
| irritable (gereizt) | .800 | .400 | .837 | .548 | 2.138 | 1.633 | .099 | .178 |
| ashamed (beschämt) | 1.800 | 1.800 | 1.304 | 1.483 | 3.087 | 2.714 | .037 | .053 |
| nervous (nervös) | .200 | -.400 | 1.924 | 1.517 | .232 | -.590 | .828 | .587 |
| jittery (durcheinander) | .200 | .200 | 1.304 | 2.168 | .343 | .206 | .749 | .847 |
| afraid (ängstlich) | .400 | .400 | .894 | .548 | 1.000 | 1.633 | .374 | .178 |
|  |  |  |  |  |  |  |  |  |

Note. N = 5.

The values are generated by subtracting posttest from pretest values.

PANAS = Positive And Negative Affect Schedule.

**Results from questionnaires in the final study.**

**Table 6**

*Mean and Standard Deviation From the EES-D Questionnaire*

| EES-D |  | *M* | *SD* |
| --- | --- | --- | --- |
| curious (neugierig) | pre | 3.935 | 0.964 |
|  | post | 2.677 | 1.107 |
|  |  |  |  |
| surprised (überrascht) | pre | 1.613 | 0.715 |
|  | post | 2.677 | 1.013 |
|  |  |  |  |
| confused (verwirrt) | pre | 1.355 | 0.551 |
|  | post | 1.742 | 0.815 |
|  |  |  |  |
| anxious (ängstlich) | pre | 1.355 | 0.798 |
|  | post | 1.484 | 0.851 |
|  |  |  |  |
| frustrated (frustriert) | pre | 1.419 | 0.672 |
|  | post | 2.355 | 1.142 |
|  |  |  |  |
| excited (begeistert) | pre | 2.903 | 0.978 |
|  | post | 1.677 | 0.909 |
|  |  |  |  |
| bored (gelangweilt) | pre | 1.516 | 0.769 |
|  | post | 1.387 | 0.558 |
|  |  |  |  |

Note. N = 31.

EES-D = Epistemically-Related Emotion Scale.

**Table 7**

*Results from the t-Test From the EES-D Questionnaire*

| EES-D | *t*(30) | *p* | Cohen's *d* |
| --- | --- | --- | --- |
| curious (neugierig) | -4.956 | < .001 | -0.890 |
| surprised (überrascht) | 4.343 | < .001 | 0.780 |
| confused (verwirrt) | 2.834 | 0.008 | 0.509 |
| anxious (ängstlich) | 0.849 | 0.403 | 0.152 |
| frustrated (frustriert) | 4.212 | < .001 | 0.757 |
| excited (begeistert) | -6.892 | < .001 | -1.238 |
| bored (gelangweilt) | -0.941 | 0.354 | -0.169 |
|  |  |  |  |

Note. Student's t-test.

N = 31.

The values are generated by subtracting posttest from pretest values.

EES-D = Epistemically-Related Emotion Scale.

**Table 8**

*Mean and Standard Deviation From the PANAS Questionnaire*

| PANAS |  | *M* | *SD* |
| --- | --- | --- | --- |
| active (aktiv) | pre | 3.290 | 0.902 |
|  | post | 2.710 | 0.973 |
|  |  |  |  |
| interested (interessiert) | pre | 4.032 | 0.836 |
|  | post | 3.548 | 0.961 |
|  |  |  |  |
| excited (freudig erregt) | pre | 2.710 | 1.006 |
|  | post | 1.548 | 0.768 |
|  |  |  |  |
| strong (stark) | pre | 2.839 | 0.934 |
|  | post | 2.161 | 1.036 |
|  |  |  |  |
| inspired (angeregt) | pre | 2.355 | 0.877 |
|  | post | 2.645 | 0.985 |
|  |  |  |  |
| proud (stolz) | pre | 2.065 | 0.892 |
|  | post | 1.710 | 0.902 |
|  |  |  |  |
| enthusiastic (begeistert) | pre | 2.839 | 1.068 |
|  | post | 1.613 | 0.955 |
|  |  |  |  |
| alert (wach) | pre | 3.516 | 0.962 |
|  | post | 3.419 | 0.807 |
|  |  |  |  |
| determined (entschlossen) | pre | 3.226 | 0.884 |
|  | post | 2.806 | 0.980 |
|  |  |  |  |
| attentive (aufmerksam) | pre | 3.806 | 0.749 |
|  | post | 3.387 | 0.844 |
|  |  |  |  |
| distressed (bekümmert) | pre | 1.548 | 0.850 |
|  | post | 3.000 | 1.065 |
|  |  |  |  |
| upset (verärgert) | pre | 1.129 | 0.341 |
|  | post | 3.194 | 1.195 |
|  |  |  |  |
| guilty (schuldig) | pre | 1.194 | 0.477 |
|  | post | 2.548 | 0.995 |
|  |  |  |  |
| scared (erschrocken) | pre | 1.065 | 0.250 |
|  | post | 3.129 | 1.384 |
|  |  |  |  |
| hostile (feindselig) | pre | 1.065 | 0.250 |
|  | post | 2.000 | 1.065 |
|  |  |  |  |
| irritable (gereizt) | pre | 1.258 | 0.445 |
|  | post | 1.935 | 0.929 |
|  |  |  |  |
| ashamed (beschämt) | pre | 1.129 | 0.341 |
|  | post | 2.806 | 1.223 |
|  |  |  |  |
| nervous (nervös) | pre | 1.935 | 0.892 |
|  | post | 1.484 | 0.811 |
|  |  |  |  |
| jittery (durcheinander) | pre | 1.484 | 0.677 |
|  | post | 2.097 | 0.870 |
|  |  |  |  |
| afraid (ängstlich) | pre | 1.194 | 0.601 |
|  | post | 1.387 | 0.844 |
|  |  |  |  |

Note. N = 31.

PANAS = Positive And Negative Affect Schedule.

**Table 9**

*Results from the t-Test From the PANAS Questionnaire*

| PANAS | t(30) | p | Cohen's *d* |
| --- | --- | --- | --- |
| active (aktiv) | -3.374 | 0.002 | -0.606 |
| interested (interessiert) | -2.802 | 0.009 | -0.503 |
| excited (freudig erregt) | -6.445 | < .001 | -1.158 |
| strong (stark) | -3.851 | < .001 | -0.692 |
| inspired (angeregt) | 1.201 | 0.239 | 0.216 |
| proud (stolz) | -2.079 | 0.046 | -0.373 |
| enthusiastic (begeistert) | -6.892 | < .001 | -1.238 |
| alert (wach) | -0.619 | 0.540 | -0.111 |
| determined (entschlossen) | -2.277 | 0.030 | -0.409 |
| attentive (aufmerksam) | -2.530 | 0.017 | -0.454 |
| distressed (bekümmert) | 6.857 | < .001 | 1.232 |
| upset (verärgert) | 8.915 | < .001 | 1.601 |
| guilty (schuldig) | 7.409 | < .001 | 1.331 |
| scared (erschrocken) | 8.578 | < .001 | 1.541 |
| hostile (feindselig) | 4.902 | < .001 | 0.880 |
| irritable (gereizt) | 3.851 | < .001 | 0.692 |
| ashamed (beschämt) | 7.822 | < .001 | 1.405 |
| nervous (nervös) | -2.447 | 0.020 | -0.439 |
| jittery (durcheinander) | 2.979 | 0.006 | 0.535 |
| afraid (ängstlich) | 2.257 | 0.031 | 0.405 |
|  |  |  |  |

Note. Student's t-test.

N = 31.

Values are generated by subtracting posttest from pretest values.

PANAS = Positive And Negative Affect Schedule.

**Table 10**

*Descriptive Data From all Self-Report Measures*

| Measure | | No. of items | Min. | Max. | *M* | *SD* | Cronbach’s α |
| --- | --- | --- | --- | --- | --- | --- | --- |
| PANAS^c^ | positive affect | 10 | 2.07 ^a^ | 4.03 ^a^ | 3.07 ^a^ | .59 ^a^ | .843 ^a^ |
|  |  |  | 1.55 ^b^ | 3.55 ^b^ | 2.56 ^b^ | .58 ^b^ | .832 ^b^ |
|  | negative affect | 10 | 1.07 ^a^ | 1.94 ^a^ | 1.30 ^a^ | .31 ^a^ | .747 ^a^ |
|  |  |  | 1.39 ^b^ | 3.20 ^b^ | 2.36 ^b^ | .73 ^b^ | .877 ^b^ |
| EES-D^c^ | curious,  surprised,  excited | 3 | 1.61 ^a^ | 3.94 ^a^ | 2.82 ^a^ | .64 ^a^ | .534 ^a^ |
|  |  |  | 1.68^b^ | 2.68^b^ | 2.34^b^ | .56^b^ | -.130^b^ |
|  | confused,  anxious,  frustrated,  bored | 4 | 1.36^a^ | 1.52^a^ | 1.41^a^ | .486^a^ | .632^a^ |
|  |  |  | 1.39^b^ | 2.36^b^ | 1.74^b^ | .59^b^ | .622^b^ |
| AEQ^c^ | enjoy, pride, hope | 13 | 3.17^b^ | 3.23^b^ | 3.20^b^ | .71^b^ | .847^b^ |
|  | anger, anxiety,  shame,  hopelessness,  boredom | 32 | 1.74 ^b^ | 2.521 ^b^ | 2.12 ^b^ | .64 ^b^ | .868 ^b^ |
| RS |  | 13 | 4.68 ^a^ | 5.9 ^a^ | 5.23 ^a^ | .35 ^a^ | .687 ^a^ |
| Learning performance^d^ | | 11 | 6^a^ | 18^a^ | 11.71^a^ | 3.08^a^ | - |
|  |  |  | 17^b^ | 26^b^ | 21.71^b^ | 2.48^b^ | - |

*Note.* N = 31.

^a^pretest, ^b^posttest. ^c^itemized by valence (positive & negative). ^d^maximum score = 33.

PANAS = Positive And Negative Affect Schedule.

EES-D = Epistemically-Related Emotion Scale.

AEQ = Academic Emotions Questionnaire.

RS = Resilience Scale.

Values are generated by subtracting posttest from pretest values.

**Table 11**

*Mean and Standard Deviation From the AEQ Questionnaire*

| AEQ | *M* | *SD* |
| --- | --- | --- |
| enjoy | 3.226 | .699 |
| hope | 3.172 | .788 |
| pride | 3.210 | .938 |
| anger | 1.916 | .858 |
| anxiety | 2.521 | .836 |
| shame | 2.221 | .737 |
| hopelessness | 1.742 | .749 |
| boredom | 2.190 | .755 |
|  |  |  |

Note. N = 31.

AEQ = Academic Emotions Questionnaire.

**Table 12**

*Mean and Standard Deviation From the Questionnaire About Prior Knowledge (Pre) and After Learning (Post)*

| Learning Outcome | *M* | *SD* |
| --- | --- | --- |
| Pre | 11.710 | 3.079 |
| Post | 21.710 | 2.479 |

Note. N = 31.

Max. score = 33.

**Table 13**

*Results From the t-Test From the Questionnaire About Learning*

|  | *t*(30) | *p* | Cohen's *d* |
| --- | --- | --- | --- |
| Learning Outcome | 13.172 | < .001 | 2.366 |

Note. Student's t-test.

N = 31.

Values are generated by subtracting posttest from pretest values.

**Table 14**

*Prototypical Items From all Self-Report Measures*

| Scale | Prototypical Item | |
| --- | --- | --- |
|  | English | German |
| PANAS | “active”  Likert-scale:  1 = very slightly or not at all  2 = a little  3 = moderately  4 = quite a bit  5 = extremely | „aktiv“  1 = ganz wenig oder gar nicht  2 = ein bisschen  3 = einigermaßen  4 = erheblich  5 = äußerst |
| EES-D | “curious”  1 = not at all,  2 = very little,  3 = moderate,  4 = strong,  5 = very strong | „neugierig“  1 = gar nicht,  2 = ein bisschen,  3 = teilweise,  4 = ziemlich,  5 = sehr |
| AEQ | Enjoyment: “I enjoy the challenge of learning the material.”  Boredom: “The material bores me so much that I feel depleted.” | Freude: “Ich genieße die Herausforderung, den Stoff zu lernen.“  Langeweile: „Der Stoff langweilt mich so sehr, dass ich mich ausgelaugt fühle.“ |
| RS | I can also get myself to do things that I don't really want to do. | Ich kann mich auch überwinden, Dinge zu tun, die ich eigentlich nicht machen will. |
|  |  |  |

*Note.* PANAS = Positive And Negative Affect Schedule.

EES-D = Epistemically-Related Emotion Scale.

AEQ = Academic Emotions Questionnaire.

RS = Resilience Scale.
